# Enhanced Production of the Mical Redox Domain for Enzymology and F-actin Disassembly Assays

**DOI:** 10.3390/ijms22041991

**Published:** 2021-02-17

**Authors:** Jimok Yoon, Heng Wu, Ruei-Jiun Hung, Jonathan R. Terman

**Affiliations:** 1 Departments of Neuroscience and Pharmacology, The University of Texas Southwestern Medical Center, Dallas, TX 75390, USA; ystar81@gmail.com (J.Y.); Heng.Wu@UTSouthwestern.edu (H.W.); rj.lindahung@gmail.com (R.-J.H.); 2 Neuroscience Graduate Program, The University of Texas Southwestern Medical Center, Dallas, TX 75390, USA

**Keywords:** oxidoreductase, F-actin disassembly, repulsion, repellent, semaphorin, plexin, MICAL1, MICAL2, MICAL3, *Drosophila*

## Abstract

To change their behaviors, cells require actin proteins to assemble together into long polymers/filaments—and so a critical goal is to understand the factors that control this actin filament (F-actin) assembly and stability. We have identified a family of unusual actin regulators, the MICALs, which are flavoprotein monooxygenase/hydroxylase enzymes that associate with flavin adenine dinucleotide (FAD) and use the co-enzyme nicotinamide adenine dinucleotide phosphate (NADPH) in Redox reactions. F-actin is a specific substrate for these MICAL Redox enzymes, which oxidize specific amino acids within actin to destabilize actin filaments. Furthermore, this MICAL-catalyzed reaction is reversed by another family of Redox enzymes (SelR/MsrB enzymes)—thereby revealing a reversible Redox signaling process and biochemical mechanism regulating actin dynamics. Interestingly, in addition to the MICALs’ Redox enzymatic portion through which MICALs covalently modify and affect actin, MICALs have multiple other domains. Less is known about the roles of these other MICAL domains. Here we provide approaches for obtaining high levels of recombinant protein for the Redox only portion of Mical and demonstrate its catalytic and F-actin disassembly activity. These results provide a ground state for future work aimed at defining the role of the other domains of Mical — including characterizing their effects on Mical’s Redox enzymatic and F-actin disassembly activity.

## 1. Introduction

**Figure 1:**
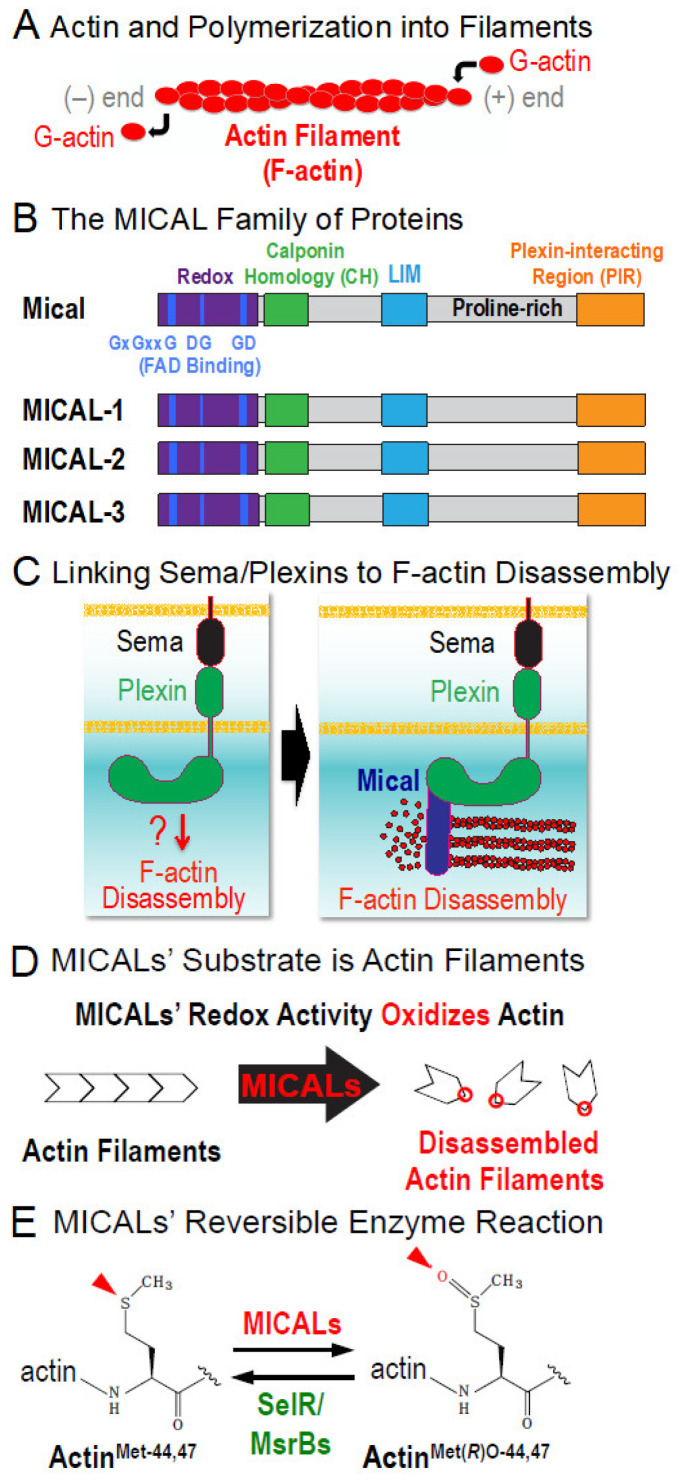
The MICALs are a family of F-actin modifying and dismantling enzymes. (**A**) Single actin proteins (G-actin) form filaments (F-actin) to control numerous cellular behaviors and tissue functions. (**B**) The MICAL family of proteins are oxidoreductase enzymes. MICALs are conserved from invertebrates (e.g., *Drosophila* Mical) to vertebrates (MICAL-1, MICAL-2 and MICAL-3, which are coded for by 3 different genes). In addition to their Redox enzymatic region (purple), which have 3 conserved motifs for binding flavin adenine dinucleotide (FAD) (blue), MICALs have multiple other domains including a calponin homology (CH) domain (green), a LIM domain (cyan), a stretch of proline residues that serve as ligands for SH3-domain containing proteins (proline-rich) and a coiled-coil C-terminal region that binds the Semaphorin receptor Plexin (Plexin-interacting Region, orange), as well as Rab family small GTPases. (**C**) MICALs provide a means by which one of the largest protein families of extracellular cues, the Semaphorins (Sema) and their Plexin receptors, disassemble F-actin. (**D**) F-actin serves as a substrate for MICALs—such that the MICALs are activated by F-actin and then MICALs oxidize and disassemble actin filaments. In particular, Mical oxidizes the methionine (Met)-44 and 47 residues of actin (to form Met-44,47-sulfoxide). Met44 & 47 are located at the pointed end of actin where monomers (subunits) join to form a filament and their oxidation by Mical induces F-actin structural changes that trigger them to disassemble [13,14,15,16,19,20,25]. (**E**) Summary of Mical’s enzymatic reaction showing that individual actin subunits become stereospecifically (*R*) oxidized (O) on their Met44 & 47 residues by Mical (forming Actin^Met(*R*)O-44,47^). These effects of Mical on actin are reversed by SelR/MsrB methionine sulfoxide reductases.

From development, and throughout maturity, a normal functioning organism requires its cells to undergo changes to their form and function. The means by which cells are able to perform these changes is through the ability of actin and tubulin proteins to come together to form actin filaments (F-actin) and microtubules (MTs), respectively [1,2]. Indeed, it is the dynamic nature of F-actin and MTs—their assembly and disassembly dynamics—that provides structure to cells and underlies their abilities to change shape, move, connect and accomplish a myriad of other behaviors [1,2]. For example, with regards to actin filaments (Figure 1A), small molecules such as Mg^2+^ and ATP modulate actin polymerization and specific proteins physically associate and use non-covalent mechanisms to control actin [1]. Although less-well appreciated, F-actin is also regulated through the covalent alteration of its amino acids and characterizing these modifications constitutes a critical direction for further study [3,4].

While investigating the molecular mechanisms that specify cellular shape, motility and navigation [5,6,7], we have characterized a novel protein family, the MICALs, that are similar at the amino acid level to oxidation-reduction (Redox) enzymes (Figure 1B; [8,9]). MICALs are conserved from invertebrates to humans—with three in mammals including humans (called MICAL-1, MICAL-2 and MICAL-3) and one in invertebrates such as flies (called Mical) (Figure 1B; [8]). Further work revealed that the MICALs are critical for cellular shape, motility and navigation (reviewed in [9,10,11,12])—and provide a direct link between one of the largest families of extracellular cues, the Semaphorins and their Plexin cell surface receptors and the modification of the actin cytoskeleton (Figure 1C; [8,13]). While deciphering the role of the interaction between Mical and actin filaments, we discovered a regulated Redox biochemical reaction mechanism through which the assembly and disassembly of actin is controlled [13,14]. Specifically, actin (F-actin) serves as a substrate for the Redox domain of Mical (Figure 1D; [13,14])—such that Mical’s Redox domain and activity selectively oxidizes specific actin methionine residues (Met-44 and Met-47) to disassemble filaments (Figure 1D,E; [14,15,16]). Furthermore, a specific enzyme SelR/MsrB reverses Mical-mediated methionine oxidation and counteracts Mical’s actin regulatory effects (Figure 1E; [15,17]). Moreover, this new biochemical mechanism is impinged upon by specific signaling pathways (e.g., [8,18,19,20,21,22,23,24]), is conserved from insects to humans (e.g., [25]) and directs multiple fundamental biological events from neuro to cancer biology (reviewed in [9,10,11,12]).

These previous results uncover a reversible Redox biochemical mechanism and signaling system that controls F-actin dynamics and cellular/systems-level behaviors. Yet, among a number of questions aimed at further understanding this MICAL Redox activity and its biological importance, are research directions focused on better understanding the roles of the other domains of the MICALs (Figure 1B), either alone or in tandem with the Redox only portion of the MICALs. Here, we describe methods for purifying the Redox only portion of Mical. Together with previous strategies we used to purify other parts of Mical, these approaches provide important starting points for future work deciphering the roles that the different MICAL domains play in affecting Mical’s F-actin-triggered Redox enzymatic activity and its effects on F-actin disassembly.

## 2. Results

MICAL family enzymes have recently emerged as critical regulators of cytoskeletal remodeling—with effects in multiple different types of cells and tissues in vivo (reviewed in [9,10,11,12]). Complementing this in vivo work has been in vitro assays with purified proteins that have revealed much about how the MICALs exert their effects on the cytoskeleton (reviewed in [9,10,11,12]). In particular, the MICALs are oxidoreductase (Redox) enzymes that bind and utilize F-actin as a substrate—such that F-actin triggers the MICALs Redox enzymatic activity and MICALs then oxidize two specific methionine residues on actin to destabilize and disassemble actin filaments (Figure 1D,E; [13,14,25]; reviewed in [9,10,11,12]). Notably, in addition to their Redox enzymatic portion (which is located at their *N*-terminus), MICALs contain multiple other domains—such that proceeding *N*-terminally to *C*-terminally is a calponin homology (CH) domain, a LIM domain, a stretch of proline residues that serve as ligands for SH3-domain containing proteins and a coiled-coil region that binds the Semaphorin receptor Plexin, as well as Rab family small GTPases (Figure 1B; [8]; reviewed in [9,10,11,12]). While important progress has been made (reviewed in [9,10,11,12]), the roles of these other MICAL domains in affecting the actions of MICALs and/or the Redox enzymatic activity of MICALs is still incompletely understood. For example, our previous work has focused primarily on the characterization of the full-length Mical protein—as well as a truncated form of Mical containing only the Redox and CH domains of Mical (Mical^RedoxCH^)—because we find that this RedoxCH portion of Mical is both necessary and sufficient in vivo and in vitro for carrying-out Mical’s effects (e.g., [13,14,15,16,19,20,25]). Notably, this work has been significantly aided by our development of simple, high-throughput approaches to express and purify the Mical^RedoxCH^ protein [13,26,27]. Yet, we have found that these approaches do not work well for expressing and purifying large amounts of other truncated forms of Mical such as the Redox only portion of Mical. Obtaining large amounts of the Redox only portion of Mical protein would provide a critical baseline/ground state for examining the role of the other domains of Mical in high-end biochemical, catalytic, imaging and structural applications. Therefore, the objectives of this study were to: (1) establish a system to express high amounts of soluble Mical^Redox^ protein, (2) develop a simple and efficient chromatography approach to purify large amounts of Mical^Redox^ protein, (3) demonstrate the purified Mical^Redox^ protein’s authenticity with regards to enzymology by performing enzyme assays (nicotinamide adenine dinucleotide phosphate (NADPH) co-enzyme consumption activity assays) and (4) determine that the purity and catalytic efficiency of the purified Mical^Redox^ protein are sufficient for characterizing Mical and its effects on actin filaments.

### 2.1. Cold Adapted Chaperonins Alone Do Not Work Well for Expressing High-Levels of Mical^Redox^ Protein

Our work with MICALs and efforts to express and purify MICAL proteins—including attempts utilizing different prokaryotic and eukaryotic expression systems—was initially confounded by both low solubility and yield [8,26]. However, our further work revealed that using a bacterial system and lowering the temperature of growth resulted in more soluble Mical protein [13,26,27]. Moreover, turning to bacterial cells engineered to express cold-adapted chaperonin proteins Cpn60 and co-chaperonin Cpn10 from the cryophilic bacterium, *Oleispira antarctica* [28] and using induction temperatures as low as 4–12 °C revealed substantially more soluble Mical protein and could be used as a platform to obtain high levels of Mical protein [27]. This low-temperature expression system has proven critical for our current understanding of Mical and its mechanisms of action [13,14,15,16,19,20,25], including that that the Redox and CH domains of Mical are together sufficient for Mical’s effects on actin filaments in vitro and in vivo [13]. The expression and purification strategies that we developed [27] have also proven critical for obtaining high amounts of RedoxCH portion of the three human MICAL family members, which allowed the comparison of the different MICAL family members from invertebrates to humans [25]. In contrast, when we used this low-temperature expression strategy to purify the Redox only part of Mical (without the CH domain), we found that the low-temperature system and strategy that we had used to express and purify the RedoxCH portions of the MICALs did not work well for expressing and purifying the Redox portion only (e.g., Figure S1A,B). We, therefore, sought to couple the low temperature expression system with other strategies to increase the production of Mical^Redox^ protein.

### 2.2. The Use and Removal of Solubility Tags Destabilizes Mical^Redox^ Protein

The addition of soluble fusion protein partners is widely used and successful in increasing protein solubility in *Escherichia coli* (*E. coli*) [29]. Multiple different solubility tags have been successfully employed, although their ability to increase solubility varies depending on the target protein of interest [29]. Our results have revealed that a Nus solubility tag [30,31,32,33], in contrast to other hydrophilic solubility tags such as glutathione-S-transferase (GST) and maltose-binding protein (MBP), greatly increased the solubility of Mical^RedoxCH^ protein [8,26,27]. Furthermore, by engineering a modified Nus solubility tag expressing vector (called pET43.1bNG; [34]) and expressing Mical^RedoxCH^ protein using this bacterial expression vector, we found that (1) Mical^RedoxCH^ protein was even more soluble in this Nus tag configuration and (2) when using Nickel (Ni^2+^)-affinity chromatography, the position of the Ni^2+^-binding His tags in this new expression vector led to an increase in the amount of Mical^RedoxCH^ protein that bound to the Ni^2+^ column [27]. We therefore used this solubility tag strategy, coupled with a low-temperature expression system, to purify large amounts of the RedoxCH domains of both the *Drosophila* and human MICAL proteins [25,27].

**Figure 2:**
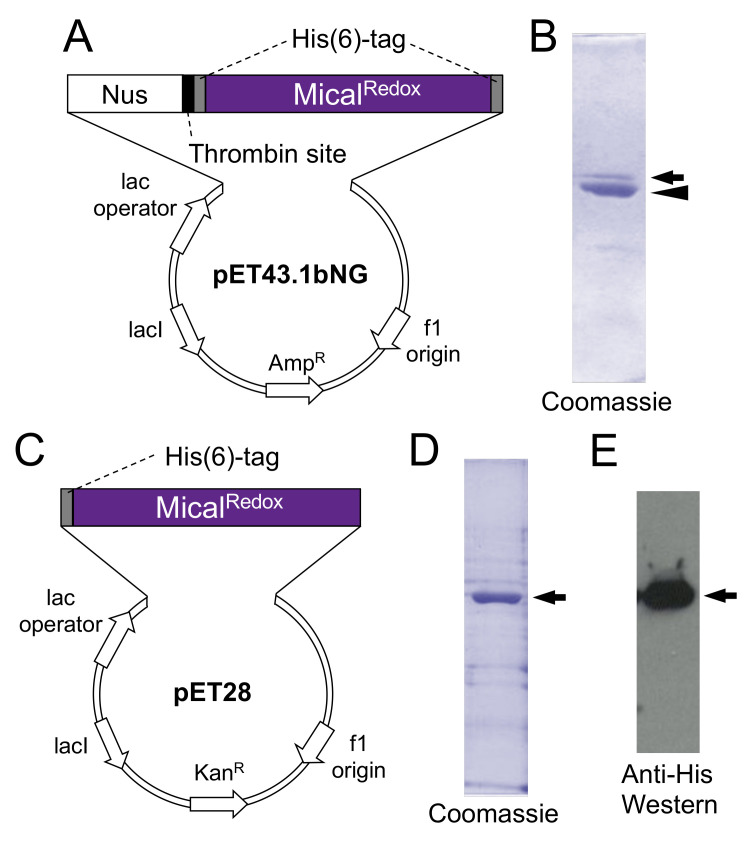
Expression and purification of Mical^Redox^ protein with different fusion partners and chaperonins. (**A**,**B**) Nus solubility tag and low-temperature chaperonins only allow expression and solubility of small amounts of the Redox only portion of Mical. (**A**) Vector (pET43.1bNG) and insert for expressing a Nus solubility tag fused to Mical^Redox^ protein. (**B**) Following thrombin digestion to remove the Nus solubility tag, the expected 1:1 ratio of Nus:Mical^Redox^ protein is affected. In particular, while the Nus tag remains soluble (arrowhead), little Mical^Redox^ protein remains in the soluble fraction (arrow). (**C**–**E**) Low-temperature chaperonins in combination with no solubility tags allow high expression and solubility of the Redox only portion of Mical. (**C**) Vector (pET28) and insert for expressing Mical^Redox^ protein without a solubility tag. (**D**,**E**) High levels of Mical^Redox^ protein is expressed and is soluble (arrows) with this expression strategy, as can be seen by Coomassie staining (**D**) and His antibody Western analysis (**E**).

We were also hopeful that this Nus solubility tag purification strategy would prove useful for purifying Mical^Redox^ protein. However, we found that while the Nus solubility tag was effective for expressing and purifying small amounts of the Redox only portion of Mical either without [26] or with the use of the low-temperature expression system (Figure 2A,B and Figure S1A,B), this strategy was not effective at expressing and purifying large amounts of the Redox only part of Mical. In particular, our results suggested that when we used a protease to remove the Nus solubility tag, this destabilized the Redox only part of Mical (Figure 2B). We therefore turned to other strategies to increase the expression and solubility of Mical^Redox^ protein. Our previous work examining the expression of the Redox only part of Mical using multiple different bacterial expression vectors (and tags) including pGEX-4T, pGEX-KG, pET25, pET28, pET30 and pET43.1/pET43.1bNG, revealed that only expression with the pET43.1/pET43.1bNG (Nus solubility tag) and pET28 (no solubility tag) vectors gave us soluble Mical^Redox^ protein [8,26]. Since our use of the pET43.1/pET43.1bNG vector (and its Nus solubility tag) was not supportive of expression of high-levels of soluble Mical^Redox^ protein (as described above), we examined the use of the pET28 vector, which does not have a solubility tag. We found that we could obtain low levels of expression and solubility of Mical^Redox^ protein when expressed using the pET28 vector (Figure 2C; [26]). We, therefore, utilized this vector to see if we could optimize and increase the solubility and production of the Mical^Redox^ protein.

### 2.3. Enhanced Expression and Solubility of Mical^Redox^ Protein Using Low-Temperature Expression, Chaperonins and No Solubility Tag

Given that we found that we also obtained low levels of expression and solubility of Mical^Redox^ protein when using cold-adapted chaperonin proteins Cpn60 and co-chaperonin Cpn10, we wondered if combining the use of the pET28 vector (no solubility tag) and cold-adapted chaperonins might increase the expression and solubility of the Mical^Redox^ protein. Therefore, *Mical^Redox^* in the pET28a plasmid was transformed into ArcticExpress^TM^ competent cells (containing the cold-adapted chaperonin proteins Cpn60 and co-chaperonin Cpn10) and the transformed bacteria were plated on antibiotic for overnight incubation at 37 °C. A single antibiotic-resistant colony was then cultured as described in detail in Section 4.2 of the Materials and Methods. Then, using the low temperature induction system (making use of the cold-adapted chaperonin proteins within the ArcticExpress^TM^ bacteria), the culture was cooled to 10 °C and the expression of Mical^Redox^ protein was induced by adding Isopropyl-β-D-thiogalactoside (IPTG) into the culture flasks. The culture was then incubated at 10 °C on a shaker at high-speed for 24 h. Lysing the bacterial and electrophoresing the lysates on a sodium dodecyl sulfate-polyacrylamide gel electrophoresis (SDS-PAGE) gel revealed that using low-temperature expression/induction, cold-adapted chaperonin proteins and the pET28 vector resulted in an increase in the levels of soluble Mical^Redox^ protein as judged using Coomassie staining and Western blotting (Figure 2C–E). Therefore, we set out to develop a system to optimally purify the soluble Mical^Redox^ protein. One such approach, detailed below (Section 2.4), provides a simple two-step purification process to obtain highly pure Mical^Redox^ protein for enzymatic and actin disassembly assays (Section 2.5 and Section 2.6).

### 2.4. Simple Two-Step Purification Strategy for Obtaining Mical^Redox^ Protein

**Figure 3:**
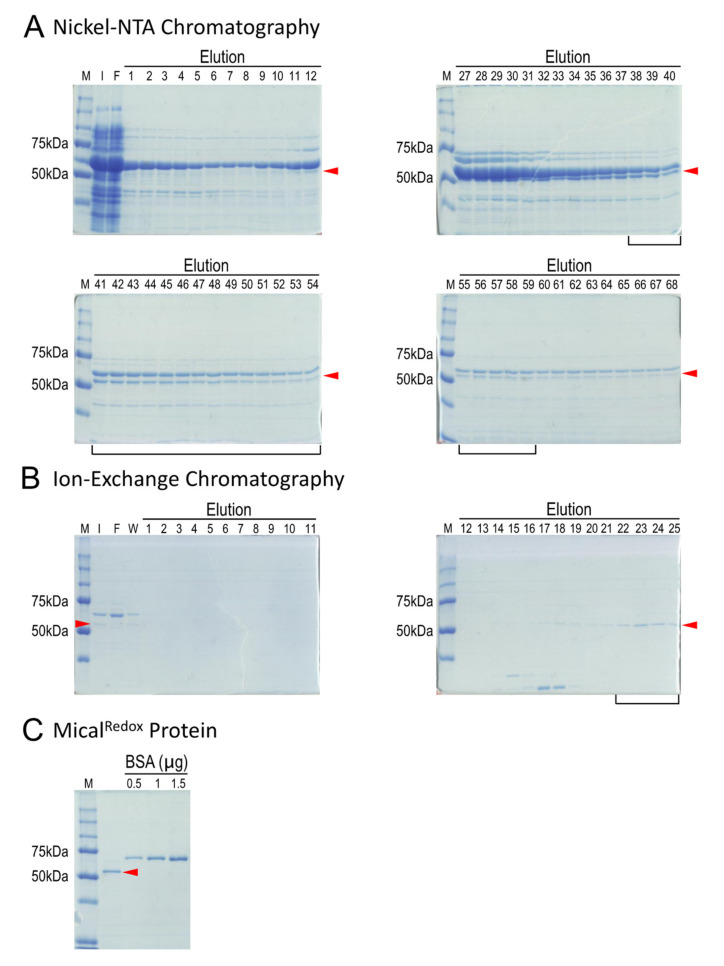
Purification of recombinant Mical^Redox^ protein. Coomassie stained bands are shown and the arrowheads point to the His(6)-tagged Mical^Redox^ protein in all gels. (**A**) Mical^Redox^ protein (arrowhead) eluted from Nickel columns was observed in multiple fractions. Cpn60 can also be seen (large band above the Mical^Redox^ protein). The fractions containing Mical^Redox^ protein (e.g., 11–68) were combined to load on to an ion-exchange column. In this particular purification, only fractions 38–59 (black bracket) were combined and used for loading on the ion-exchange column. (**B**) A strong cation exchange Mono S column was used to separate Mical^Redox^ protein (arrowhead) from other contaminating proteins including Cpn60. Fractions enriched with Mical^Redox^ protein (22–25; black bracket) were combined and the buffer system was changed to the storage buffer. (**C**) Mical^Redox^ protein (arrowhead) and purity were checked by running on a gel next to known concentrations of bovine serum albumin (BSA) protein. M, protein markers; I, input; F, flow-through; W, wash.

To purify Mical^Redox^ protein, we found it important to keep all buffers cold, which involved chilling all buffers to 4 °C and performing the purification with a fast protein liquid chromatography (FPLC) system equilibrated to 4 °C (i.e., using a cold room or cold box). We monitored Mical^Redox^ protein purity by SDS-PAGE and Coomassie blue staining as shown in Figure 3.

To begin the Mical^Redox^ protein purification process, we resuspended the bacterial pellets from above (Section 2.3) into 100 mL of cold lysis buffer and the bacteria were lysed at 4 °C with sonication. The cloudy bacterial lysates were then cleared by centrifugation at 4 °C and the supernatant was transferred into new centrifuge tubes and centrifuged again for 1 h at 4 °C. The supernatant was then collected and filtered through a 0.45 µm membrane filter. We then used Ni^2+^-NTA chromatography for the first step in purifying Mical^Redox^ protein and to begin this step we equilibrated two connected 5 mL Ni^2+^ columns with Ni-A buffer. Using an FPLC system, the filtered supernatant was then loaded into the pre-equilibrated Ni^2+^ columns using a 150 mL Superloop and the columns were washed with Ni-A buffer. In some cases, if the UV chromatogram at 280 nm that we used to monitor for the presence of protein did not return to the baseline after washing, the columns were subjected to additional washing with Ni-A buffer. The bound protein was then eluted from the Ni^2+^ columns using Ni-A buffer and Ni-B buffer with a linear gradient to 100% of Ni-B buffer. All the eluted protein was collected in 1 mL aliquots in microcentrifuge tubes and each of the fractions were run on an SDS-PAGE gel (Figure 3A). We found that Mical^Redox^ protein had a relatively high affinity to the column and was eluted at higher Ni-B concentrations (higher imidazole concentrations) (Figure 3A). Note that Mical^Redox^ protein (57 kDa) will migrate at slightly above 50 kDa (Figure 3A). Note also that cold-adapted chaperonin Cpn60 (it runs slightly higher than 60 kDa on an SDS-PAGE gel) has a similar molecular weight as Mical^Redox^ protein (57 kDa) and so gels were run long enough to separate these two proteins (Figure 3A).

The fractions that contained an abundance of Mical^Redox^ protein were then combined, desalted and concentrated to 500 µL using 50 kDa molecular weight cut-off concentrators. We then turned to ion-exchange chromatography to further purify Mical^Redox^ protein. In particular, this concentrated fraction of Mical^Redox^ protein was first diluted/transferred into S-A buffer. Notably, although we did not quantify it, we observed that Mical^Redox^ protein became insoluble in low salt concentrations (<100 mM). Therefore, care was taken to maintain the salt concentration at 100 mM or above. Furthermore, the predicted isoelectric point (pI) of Mical^Redox^ protein is 8.4 and Mical^Redox^ protein will be positively charged in the S-A buffer system (pH6.5) (see below). Therefore, to ensure that Mical^Redox^ protein binds to the ion-exchange (MonoS) column, we found that it was important to check the pH of the solution before loading the Mical^Redox^ protein sample onto the Mono S column. After this was confirmed, the protein sample containing the Mical^Redox^ protein in S-A buffer was then loaded into the pre-equilibrated ion exchange Mono S column at 0.3 mL/min flow rate using an FPLC system. The column was then washed with S-A buffer at 0.3 mL/min flow rate. The bound protein was then eluted from the Mono S column using S-A buffer and S-B buffer with a linear gradient to 50% of S-B buffer and then subjected to additional washes with 50% S-B buffer.

Mical^Redox^ protein was found to elute at around a conductivity of 52 ms/cm. Conductivity indicates a material’s ability to conduct electric current—where the conductivity of a solution is determined by ions (for example, the Na^+^ and Cl^−^ in S-A and S-B buffers) contained in the solution and the higher the concentration of ions, the higher the conductivity of the solution. Mical^Redox^ protein is positively charged in the S-A buffer system and binds to the MonoS column. Mical^Redox^ protein was eluted at a conductivity of 52 ms/cm, corresponding to 325 mM NaCl, and Mical^Redox^ protein can be seen in specific eluate fractions from the Mono S column (Figure 3B). Samples containing the Mical^Redox^ protein were then exchanged into a stable storage buffer (Mical^Redox^ protein storage buffer) using 50 kDa molecular weight cut-off concentrators. The concentration of the Mical^Redox^ protein was then determined using either a Bradford assay [35,36] or a spectrophotometer at 280 nm absorption. It should also be noted that, although we did not quantify it, we observed that Mical^Redox^ protein precipitates out of solution at high concentrations. Therefore, we found that it is best not to concentrate Mical^Redox^ protein over 2 mg/mL. The concentration and purity were also further analyzed by loading a small amount (≤1 µL) of the Mical^Redox^ protein, along with bovine serum albumin (BSA) standards at different concentrations, on an SDS-PAGE gel (Figure 3C). The Mical^Redox^ protein was then aliquoted, snap frozen in liquid nitrogen and stored at −80 °C. We found that it is stable and retains its activity for >1 year when stored in this way.

### 2.5. Biochemical Analysis of Purified Mical^Redox^ Protein: F-actin Triggered Catalytic Activity of Mical^Redox^

**Figure 4:**
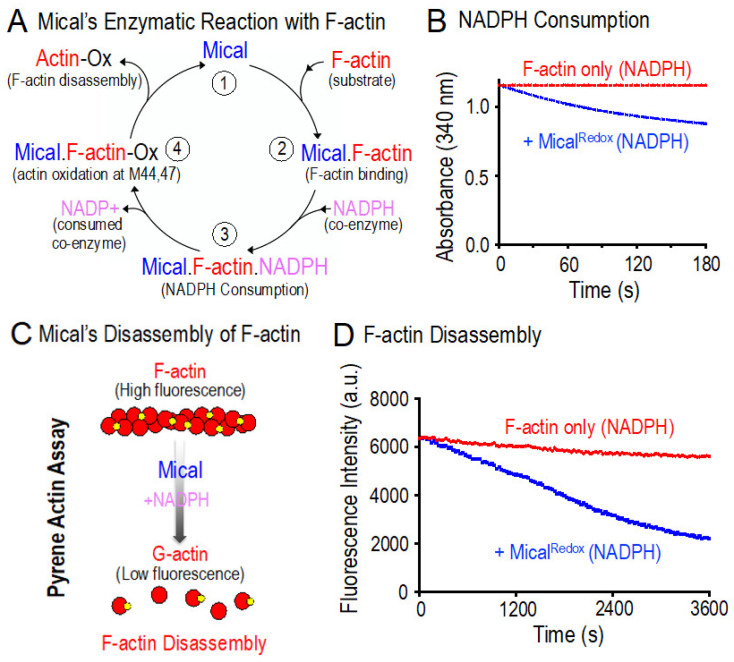
Analysis of the activity of purified Mical^Redox^ protein. (**A**) Mical’s enzymatic reaction with F-actin. This model is based on our results (reviewed in [9,10,11,12]) that Mical (1) physically associates with its substrate F-actin (2), which triggers Mical’s enzymatic activity to convert its co-enzyme nicotinamide adenine dinucleotide phosphate (NADPH) to NADP^+^ (3). Mical then oxidizes (Ox) F-actin subunits on their methionine (M) 44 and M47 residues (4), triggering F-actin disassembly. Note that for simplicity, details, such as the presence of molecular oxygen (O_2_) and conversion of flavin adenine dinucleotide (FAD) to FADH_2_ and reoxidation back FAD_,_ have been left off the model. (**B**) Mical uses the pyridine nucleotide NADPH as a co-enzyme in its Redox enzymatic reactions [13,14,15,20]. We therefore sought to confirm the enzyme activity of the purified Mical^Redox^ protein by characterizing its NADPH consumption activity. As judged by the decrease in absorbance over time (NADPH absorbs light at 340 nm, while NADP^+^ does not), our results revealed that our Mical^Redox^ protein converts NADPH to NADP^+^ and therefore has enzyme activity. Both conditions contain NADPH. [NADPH] = 200 µM; [Mical^Redox^] = 50 nM; [F-actin] = 18.4 µM. (**C**,**D**) Pyrene-labeled actin depolymerization assays demonstrate purified Mical^Redox^ protein’s ability to disassemble F-actin. (**C**) The fluorescence of pyrene-labeled actin is higher when actin is present in its polymerized form. (**D**) Similar to previous results with Mical [13,14,15,20], our newly purified Mical^Redox^ protein induces actin depolymerization in the presence of its NADPH coenzyme as judged by a Pyrene-actin depolymerization assay, where the fluorescence of polymerized actin decreases as actin depolymerizes. [Mical^Redox^] = 50 nM; [NADPH] = 100 µM; [F-actin] = 4.65 µM.

The Redox enzymatic portion of Mical regulates the biochemical properties of actin filaments (Figure 1D,E; reviewed in [9,10,11,12]). In particular, actin filaments are the building blocks of cellular form and function—providing structure to cells and the forces necessary for cell movement and reorganization [1]. In vivo work in cells and tissues reveals that Mical regulates actin filament stability and dynamics (reviewed in [9,10,11,12]). Likewise, numerous in vitro assays with purified proteins have been used to characterize Mical’s direct effects on F-actin dynamics and the mechanisms of how Mical affects these filaments (e.g., [13,14,15,16,19,20,25]). We therefore were able to make use of these assays to rapidly confirm the useability of our newly purified Mical^Redox^ protein. For example, it is critical to determine if our newly purified Mical^Redox^ protein is enzymatically active. In Mical’s enzymatic reaction with its substrate F-actin (Figure 4A (1–4)), Mical physically associates with F-actin (Figure 4A (1–2)), which triggers Mical’s association with its co-enzyme NADPH (Figure 4A (3)). Mical then consumes NADPH, catalytically converting it to NADP+ (Figure 4A (3)). We can thus use this enzyme activity of Mical to determine if our Mical^Redox^ protein is enzymatically active. Using standard assays to look at conversion of NADPH to NADP+, our results confirmed that Mical^Redox^ protein is enzymatically active and can convert NADPH to NADP+ (Figure 4B; Figure S2A–C).

### 2.6. Biochemical Analysis of Purified Mical^Redox^ Protein: F-actin Disassembly Activity of Mical^Redox^

Actin filaments are helical polymers consisting of multiple individual actin proteins (monomers (subunits)) that join together (polymerize) via the use of non-covalent bonds—and can dynamically join and leave the filament at its two ends (Figure 1A). The polymerization properties of actin can be reconstituted in vitro by using purified actin and specific constituents and conditions [37]. For example, fluorescently-labeled actins like pyrene actin provide important tools for monitoring both actin polymerization and actin depolymerization, such that the fluorescence intensity of the pyrene-labeled actin polymer is substantially higher than the pyrene-labeled actin monomer (Figure 4C; [37]). We were therefore able to employ pyrene-labeled actin to determine if our newly purified Mical^Redox^ protein has effects on F-actin stability. In particular, our previous work using pyrene actin (and multiple other assays) reveals that Mical, including Mical^Redox^ protein, uses its enzyme activity to induce F-actin disassembly (Figure 4A; [13,14,15,16,19,20,25]). Likewise, we find similar results using our newly purified Mical^Redox^ protein, such that Mical in the presence of its co-enzyme NADPH induces the disassembly of F-actin (Figure 4D and Figure S2D).

## 3. Discussion

*Escherichia coli* (*E. coli*) is the dominant host for producing recombinant proteins because of its fast, inexpensive and high-yield protein production [38]. However, because it is a prokaryotic system, *E. coli* is often inadequate at expressing eukaryotic proteins and this often results in poor solubility of the recombinant protein [38,39]. For example, studies have reported that up to 75% of the human proteins examined are expressed in *E. coli* but only 25% are produced in an active soluble form [40,41]. Numerous strategies have been developed to overcome these hurdles, however, including the use of different mutated *E. coli* strains, lowering of the culturing temperatures, co-expression of chaperones and foldases, and/or the addition of soluble fusion partners to insoluble target proteins [38,42]. Yet, the success of these strategies often varies depending on the protein of interest—and the use of particular strategies or a combination of strategies, for a given protein is often labor intensive, requiring extensive trial and error to determine the correct strategies to obtain soluble recombinant protein. However, the strategies to overcome these hurdles are typically highly effective and are therefore well worth the time and energy commitment. Here, we made use of these strategies, including employing a heterologous bacterial expression system, empirically-testing and deriving formulations for enhancing Mical enzyme generation and combining several of these different approaches to develop a simple and rapid system for substantially increasing the production of the Redox part of the Mical enzyme.

MICAL family enzymes have emerged as important actin cytoskeletal regulators—and the means by which the MICALs exert their effects on the cytoskeleton have been uncovered by coupling work in cellular/in vivo assays with work with purified Mical protein ([13,14]; reviewed in [9,10,11,12]). MICALs are unusual in that they are F-actin modifying enzymes (reviewed in [9,10,11,12]). MICALs are also unique in that they couple their actin modifying flavoprotein monooxygenase/hydroxylase (Redox) enzymatic domain with multiple other domains (reviewed in [9,10,11,12]). Yet, the roles of these other MICAL domains in affecting the Redox/F-actin disassembly activity of MICALs are incompletely understood. Therefore, in the current work we set out to develop a simple expression and purification strategy that might provide a ground state for examining the role of the other domains of Mical in Mical-mediated Redox/F-actin disassembly. In particular, our previous attempts to generate different truncated forms of Mical to study the role of the different Mical domains on Mical’s Redox/F-actin disassembly activity were hampered by problems with low solubility and protein yield [8,26]. Yet, we have recently developed simple and high-throughput approaches that have proven useful for expressing and purifying some truncated Mical proteins from bacteria [25,27]. This purification strategy has been instrumental for our ability to better understand the MICALs and their mechanisms of action [13,14,15,16,19,20,25]. However, we also found that other truncated forms of Mical, such as the Redox only portion of Mical, could not be readily purified using these strategies.

Our results now provide a simple two-step method for expressing and rapidly purifying the active Redox portion of Mical from bacteria. Specifically, we have adapted a low temperature bacterial expression system with cold-adapted chaperonin proteins to be used for the expression of Mical Redox protein. We find that this strategy increases the levels of soluble Mical Redox protein. Furthermore, we have identified a bacterial expression vector that allows for soluble Mical Redox protein—as well as enhanced stability of the soluble Mical protein. Moreover, we find that combining these two strategies allows for a compounded enhancement of soluble and stabile Mical Redox protein. Utilizing these results, we then defined a simple two-step affinity and ionic exchange purification process for the Mical Redox protein—and identified the ionic conditions that allow for better solubility, stability and capture of the Redox portion of Mical. Finally, we also find that this new purified Mical Redox protein is highly active and exhibits both strong catalytic and actin filament disassembly activity. Thus, combining this new strategy with our previous approach to purify other portions of Mical will help advance the goal of using high-precision biochemical, catalytic, imaging and structural applications to determine the role of each of Mical’s domains on Mical’s Redox-mediated enzymology and F-actin disassembly activity. In particular, specific next steps such as deciphering the roles of each of the domains of Mical, including whether they might assist (or dampen) the enzyme activity of the Mical Redox domain and/or its effects on F-actin are important future goals that will now be assisted by the enhanced production of this Mical Redox only protein and the capability to directly and systematically compare it to Mical proteins that contain additional domains. Given Mical’s catalytic activity and unusual mechanism of action, its critical role in regulating the actin cytoskeleton, its widespread expression patterns and its connection to disease (reviewed in [9,10,11,12]), further investigating this Mical Redox only protein will be instrumental to better understanding the enzymology of the MICALs and ultimately the mechanisms by which they regulate cellular form, function and dysfunction.

## 4. Materials and Methods

### 4.1. Chemicals and Chromatography Reagents

All solutions were made with ultrapure deionized water (with a sensitivity of 18 MΩ at 25 °C), analytical grade reagents and sterilized by either autoclaving or filtration. All reagents were prepared and stored at room temperature unless indicated otherwise. Basic chemicals were obtained from MilliporeSigma (Burlington, MA, USA), unless noted otherwise. All restriction enzymes were obtained from New England Biolabs (Ipswich, MA, USA). Imidazole was obtained from EMD Biosciences (MilliporeSigma, Burlington, MA, USA). Conical centrifuge tubes were obtained from Corning Life Sciences (Corning, NY, USA). Bacterial lysing was performed with a Sonicator (Q700 sonicator, Qsonica, Newtown, CT, USA). Ni^2+-^NTA Columns (HisTrap^TM^ FF), ion-exchange Mono S columns (Mono S^TM^ 5/50 GL) and FPLC (AKTA Purifier UPC 10) were from GE Healthcare Bio-Sciences Corporation (Piscataway, NJ, USA).

### 4.2. Molecular Biology and Protein Expression

To generate Mical protein containing only the Redox domain, the appropriate portion of Mical (Redox domain, amino acids 44—531) including 5′ and 3′ Bgl II sites and a stop codon at 3′ end was amplified by polymerase chain reaction (PCR). After purification of the appropriately sized PCR product, the PCR product was digested with Bgl II and inserted into the compatible BamH I sites of the pET28a expression vector (Millipore Sigma, Burlington, MA, USA). The resulting recombinant protein includes a *N*-terminal His_6_-tag [13,26] (Figure 2C). Following sequencing of the insert on both strands, the Mical^Redox^ pET28 plasmid was transformed into ArcticExpress^TM^ competent cells (Agilent Technologies, Santa Clara, CA, USA) following the manufacturer’s protocol. In brief, 100 µL of bacterial competent cells were thawed on ice in a 14 mL polypropylene round bottom tube with β-mercaptoethanol and 50 ng of Mical^Redox^ pET28a DNA was added to the mixture and incubated on ice for 30 min. The transformation mixture was then heat-shocked in a 42 °C water bath for 20 s and the mixture was then incubated on ice for 2 min. 0.9 mL of pre-heated (42 °C) Super Optimal broth with Catabolite repression (SOC) medium was then added to the transformation mixture and the mixture was incubated at 37 °C for 1 h on a shaker at high-speed. The transformed bacteria were then streaked on Luria Bertani (LB)/agar plates containing 30 µg/mL kanamycin and bacteria containing the plasmid were then selected by their ability to grow on the antibiotic. Plates could be sealed with parafilm or plastic wrap and stored at 4 °C for ~1 month prior to selecting a colony for inoculation (see below). Alternatively, a glycerol stock of the transformed bacteria could be made and stored at −80 °C and a little could be struck-out/cultured for inoculation each time.

A single clone was then selected from the culture plate using sterile technique and inoculated into 150 mL complete Terrific Broth (TB) culture medium (basic TB medium and potassium phosphate solution) containing 30 µg/mL kanamycin, 20 µg/mL gentamycin and 2 mM MgSO_4_ (Thermo Fisher Scientific, Waltham, MA, USA) and shaken at 37 °C overnight in a shaking incubator (Innova44, New Brunswick, Eppendorf, Hamburg, Germany). 1 L of complete TB medium containing 30 µg/mL kanamycin was then added to each of six 2.8 L flasks. 25 mL of the overnight starter culture was then added to each of these six flasks, mixed thoroughly and then 2 mL of Antifoam B emulsion (MilliporeSigma, Burlington, MA, USA) were added into each flask. Bacteria were then cultured at high-speed/225 rpm in a 30 °C shaker until mid-log phase (O.D. 600 between 1.0–1.8 worked best for induction). At this point, we transferred 1 mL sample of the bacteria culture into a microcentrifuge tube and centrifuged the sample for 1 min at 6000× *g* at room temperature. The supernatant was then removed and the pellet was saved at −80 °C for use in the analysis of the protein present in the sample prior to induction of Mical^Redox^ protein (see below for SDS-PAGE gel analysis). To induce expression of the Mical^Redox^ protein, IPTG was then added to the culture at a final concentration of 0.2 mM and the culture was then incubated with shaking at 10 °C for 24 h. The contents of each of the culture flasks was then poured into 1 L centrifuge bottles (Nalge Nunc International Corporation, Rochester, NY, USA), which were spun in a Beckman J-6M Induction Drive Centrifuge (Beckman Coulter, Brea, CA, USA) at 3500× *g* for 30 min at 4 °C to collect the bacteria pellet. The media/supernatant was then removed and the bacterial pellets were transferred into 50 mL conical tubes to proceed with purification. Pellets could also be frozen with liquid nitrogen at this point and stored at −80 °C for purification at a later time. It should also be noted that after induction at 10 °C and prior to centrifugation, a 1 mL sample of the bacteria culture was transferred into a microcentrifuge tube and the sample was centrifuged for 1 min at 6000× *g* at room temperature. The supernatant was then removed and the pellet was saved at −80 °C so that the protein present in the sample after induction of Mical^Redox^ protein could be analyzed (i.e., comparing the protein bands in the sample before and after induction indicates if the expression of Mical^Redox^ protein is successfully induced by IPTG; see below for SDS-PAGE gel analysis). We noticed that if the induction was not obvious, a new batch of IPTG stock solution should be made.

### 4.3. Homogenization and Clarification of Cell Lysates

Bacterial pellets from 6 L TB media culture were resuspended (by pipetting or vortexing) in 100 mL of lysis buffer (50 mM Tris-HCl, pH8.0, 500 mM NaCl, 20 mM imidazole) that had been stored at 4 °C. 10 mM β-mercaptoethanol and one tablet of Complete^®^ ethylenediaminetetraacetic acid (EDTA)-free Protease Inhibitors (Millipore Sigma, Burlington, MA, USA) was also added to the lysis buffer immediately before adding the lysis buffer to the bacterial pellets. The bacteria were then lysed with sonication (Q700 sonicator, Qsonica, Newtown, CT, USA) for 15 min at a 50 amplitude with 5 s on and 5 s off cycles. Cell lysates should be kept cold by putting them in an ice bath and stirring occasionally. The cell lysates turned more brownish and less viscous when the cells were well-lysed. The lysed bacteria were then clarified by transferring the sample into a 250 mL centrifuge bottle with a sealing cap (Nalgene, Nalge Nunc International Corporation, Rochester, NY, USA) and centrifuging the homogenized sample at 39,200× *g* for 2 h at 4 °C with a centrifuge (Beckman J2-MC, Beckman Coulter, Brea, CA, USA). The supernatant was then transferred into new centrifuge tubes and centrifuged again for 1 h at the same speed. The supernatant was then filtered using 0.45 µm filters (0.45 µm Durapore mixed cellulose membrane filters, MilliporeSigma, Burlington, MA, USA). A 100 µL sample of the supernatant was saved at this point for SDS-PAGE gel analysis (used as “input” in SDS-PAGE gel analysis, see below).

### 4.4. Ni^2+^-NTA Chromatography and Examination of Fractions

Chromatography using Nickel-nitrilotriacetic acid (Ni^2+^-NTA) was done using standard approaches and a FPLC machine (AKTA Purifier UPC 10; GE Healthcare Bio-Sciences Corporation, Piscataway, NJ, USA). In brief, two connected 5 mL HisTrapFF columns (GE Healthcare Bio-Sciences Corporation, Piscataway, NJ, USA) were equilibrated with at least 5 column volumes (approximately 50 mL) of Ni-A buffer (10 mM Tris-HCl, pH 8.0, 500 mM NaCl, 5% glycerol, 20 mM imidazole and with 10 mM β-mercaptoethanol added immediately before use), which had been stored at 4 °C. The sample (filtered supernatant) was then loaded into the pre-equilibrated 5 mL HisTrapFF columns at 1–2 mL/min flow rate using a 150 mL sample injection loop (Superloop, GE Healthcare Bio-Sciences Corporation, Piscataway, NJ, USA). At this point the flow through was also collected to check the binding efficiency of Mical^Redox^ protein to the columns (used as “flow through” in SDS-PAGE gel analysis, see below). The HisTrapFF columns were then washed with 10 column volumes of Ni-A buffer at 1–2 mL/min flow rate. We noted that in some cases, the columns needed to be washed with additional Ni-A buffer. For example, the protein eluting from the sample can be measured at 280 nm with a UV chromatogram. During the column wash, returning to baseline at 280 nm indicates that unbound proteins are removed and they are no longer eluting from the column. If absorption at 280 nm was above the baseline, this indicated that contaminating proteins were still being eluted and so the column was washed with additional Ni-A buffer. The Mical^Redox^ protein was then eluted from the column with elution buffer Ni-B (10 mM Tris-HCl, pH 8.0, 500 mM NaCl, 5% glycerol, 250 mM imidazole and with 10 mM β-mercaptoethanol added immediately before use), which had been stored at 4 °C. In particular, to better separate Mical^Redox^ protein from contaminating proteins, bound protein was eluted using a linear gradient. The linear gradient was set up by mixing Ni-A buffer (i.e., containing 20 mM imidazole) and Ni-B buffer (i.e., containing 250 mM imidazole) and gradually increasing the portion of Ni-B buffer in the mixture from 0% to 100% in 10 column volumes (50 mL). The eluates were saved in 1 mL aliquots in 1.7 mL capless microcentrifuge tubes using a FRAC-920 fraction collector (GE Healthcare Bio-Sciences Corporation, Piscataway, NJ, USA). 10 µL from each elute tube was then mixed with 3.3 µL of 4× Laemmli Sample Buffer (250 mM Tris, pH 6.8, 8% SDS, 40% glycerol, 0.032% bromophenol blue, 20% β-mercaptoethanol) and loaded onto an 1.5 mm-thick SDS-PAGE gel composed of 10% separating gel (10% Acryl and bisacryl [29:1], 375 mM Tris-HCl, pH 8.8, 0.1% SDS, 0.1% ammonium persulfate and 0.08% tetramethylethylenediamine (TEMED)) and 4% stacking gel (4% Acryl and bisacryl [29:1], 125 mM Tris-HCl, pH6.8, 0.1% SDS, 0.15% ammonium persulfate and 0.125% TEMED). Additional samples (e.g., from the input (see above) and flow through (see above)) were also run on the same set of gels. All protein electrophoresis was done using a Mini Format 1-D Electrophoresis unit (Bio-Rad, Hercules, CA, USA). The gels were then stained with Coomassie gel staining solution and Coomassie gel destaining solution was also used to decrease the background staining.

### 4.5. Ion Exchange Chromatography and Examination of Fractions

The fractions that contained an abundance of Mical^Redox^ protein were then combined and concentrated to 500 µL using 50 kDa molecular weight cut-off concentrators (Amicon Ultra centrifugal filter (Ultracel-50 kDa cutoff), MilliporeSigma, Burlington, MA, USA). This concentrated fraction was then diluted with 40 mL of S-A buffer (20 mM 2-[*N*-Morpholino]ethanesulfonic acid (MES) in which the pH had been adjusted to 6.5 by adding 2 M KOH, 150 mM NaCl and with 2 mM dithiothreitol (DTT) added immediately before use), which had been stored at 4 °C. A small aliquot of supernatant was saved for SDS-PAGE gel analysis (“2nd input”, see below). An ion exchange Mono S 5/50 GL column (GE Healthcare Bio-Sciences Corporation, Piscataway, NJ, USA) was then equilibrated with at least 10 column volumes of S-A buffer. The Mical^Redox^ protein sample diluted into S-A buffer was then loaded onto a Mono S 5/50 GL column at a flow rate of 0.3 mL/min. The flow through was then collected to check the binding efficiency of Mical^Redox^ protein to the column (“2nd flow through” to be used in SDS-PAGE gel analysis, see below). The column was then washed with 10 column volumes of S-A buffer at 0.3 mL/min flow rate. Washing continued until, as described above, the UV chromatogram 280 nm reading was stable, indicating that protein was not continuing to wash off the column. Further, in a similar strategy as described above, Mical^Redox^ protein bound to the Mono S 5/50 GL column was eluted using a linear gradient to better separate Mical^Redox^ protein from contaminating proteins. The linear gradient was set up by mixing 4 °C S-A buffer and S-B buffer (20 mM MES, pH 6.5, 1000 mM NaCl and with 2 mM DTT added immediately before use) and increasing the portion of S-B buffer in the mixture from 0% to 50% in 20 column volumes. The column was then washed with 50% S-B buffer for an additional 10 column volumes. Protein with high affinity to the column was eluted at high S-B concentration (i.e., high NaCl concentration). All eluates were saved in 1 mL aliquots in 1.7 mL microcentrifuge tubes in a fraction collector as described above. 1% of the volume of each eluted fraction (10 µL), along with the 2nd input sample and the 2nd flow through sample, were then mixed with Laemmli Sample Buffer and electrophoresed on 10% acrylamide SDS-PAGE gels. The gels were then stained with Coomassie gel staining solution.

### 4.6. Buffer Exchange, Sample Concentration, Protein Quantification and Western Blotting

Samples eluted from the ion exchange column containing the appropriately sized band (Mical^Redox^ protein) were then exchanged into cold Mical^Redox^ protein storage buffer (20 mM Tris-HCl, pH8.0, 150 mM NaCl, 5% glycerol and with 2 mM DTT added immediately before use) using molecular weight cut-off concentrators (Amicon Ultra centrifugal filter (Ultracel-50 kDa cutoff), MilliporeSigma, Burlington, MA, USA) and centrifuged for 15 min at 2623× *g* with a Beckman J-6M Induction Drive Centrifuge (Beckman Coulter, Brea, CA, USA). Centrifugation and these concentrators were also used to decrease the volume and concentrate the Mical^Redox^ protein. The concentration of the Mical^Redox^ protein was determined by adding a small amount (≤1–2 µL) to the platform of a Nanodrop spectrophotometer (Thermo Fisher Scientific, Waltham, MA, USA) and measuring the absorption at 280 nm [43]. To further confirm the identity of the Mical^Redox^ protein, Western blotting was performed using standard approaches [27,44]. In brief, 0.1–0.5 µg of purified Mical^Redox^ protein and 5 µL Precision Plus Protein Standards (Bio-Rad, Hercules, CA, USA) were loaded onto a 10% SDS-PAGE gel. After electrophoresis, the protein was transferred onto PVDF membrane (Immobilon P, MilliporeSigma, Burlington, MA, USA) and the membrane was blotted with 5% dry milk/phosphate-buffered saline (PBS) for 1 h at room temperature. A 1:2000 dilution of a Mouse 6×His tag antibody (His tag Monoclonal antibody, Novagen, MilliporeSigma, Burlington, MA, USA)/5% dry milk/PBS was then added to the membrane and incubated overnight at 4 °C. The membrane was washed 5 times with PBS, incubated in a 1:10,000 dilution of horseradish peroxidase (HRP)-conjugated, sheep anti-mouse IgG antibody (GE Healthcare Bio-Sciences Corporation, Piscataway, NJ, USA) in 5% dry milk/PBS for 1 h at room temperature and then after washing in PBS, was incubated in SuperSignal^TM^ West Pico Chemiluminescent substrate (Thermo Fisher Scientific, Waltham, MA, USA). The membrane was then exposed to BioMax Light Film (Kodak, Rochester, NY, USA).

### 4.7. Analysis of Mical^Redox^ Protein: Catalytic (NADPH Consumption) Assays

To examine the ability of the Mical^Redox^ protein to be enzymatically active and consume its co-enzyme NADPH we used standard approaches that are based on the ability of NADPH to absorb light at 340 nm, while NADP^+^ does not (e.g., [14,20,25,27]). In brief, Mical^Redox^ protein or Mical^Redox^ protein storage buffer only were incubated with NADPH after basal NADPH consumption had been measured in the presence of F-actin (18.4 µM) and NADPH (200 µM) for 3 min using a Quartz and Glass Micro Cell (Thermo Fisher Scientific, Waltham, MA, USA) at 340 nm using a fluorescence spectrophotometer (Spectra max M2; Molecular Devices, San Jose, CA, USA) at 25 °C. Then, the absorbance was monitored every 2 s at 340 nm for 3 min using the same setup. The results from the spectrophotometer were then exported. The zero points of both samples were then normalized according to the pre-read results (zero point results) and the NADPH consumption curve was presented using GraphPad Prism (San Diego, CA, USA). For measuring the consumption of Mical^Redox^ protein without its F-actin substrate and while also including different concentrations of Mical^Redox^ protein, the absorbance was monitored and presented over longer periods of time using approaches described previously [25,27].

### 4.8. Analysis of Mical^Redox^ Protein: F-actin Disassembly Assays

Standard approaches for a pyrene-labeled actin polymerization assay were employed (e.g., [13,14,15,20,25,27,37]). In brief, a fluorescence spectrophotometer (SpectraMax M2, Molecular Devices, San Jose, CA, USA) with an excitation wavelength of 365 nm and an emission wavelength of 407 nm was set up at 25 °C. A pyrene-actin stock solution (20 mg/mL pyrene-labeled actin in 5 mM Tris-HCl, pH 8.0, 0.2 mM CaCl_2_, 0.2 mM ATP, 5% sucrose and 1% dextran) was diluted to 1 mg/mL (2.3 µM) by adding 400 µL 1× G-buffer (5 mM Tris-HCl pH 8.0 and 0.2 mM CaCl_2_ and a final concentration of 200 µM ATP and 1 mM DTT was added immediately before use). The actin was then polymerized by adding 10 µL of 10× actin polymerization buffer (50 mM Tris-HCl, pH 7.5, 500 mM KCl, 20 mM MgCl_2_, 10 mM ethylene glycol-bis(beta-aminoethyl ether)-N,N,N’,N’-tetraacetic acid (EGTA), pH 8.0 and a final concentration 2 mM ATP and 5 mM DTT was added immediately before use) (0.25× final strength) and incubated at room temperature for 1 h while protecting from light. The polymerized actin was then diluted to 0.2 mg/mL by adding 1.2 mL of 1× G-buffer and mixed gently by inversion. It should be noted that this dilution initiates some F-actin disassembly due to a decrease of actin concentration. The rate of this spontaneous F-actin disassembly is the basal level of F-actin disassembly in the assays. 200 µL of this actin solution was then transferred into each well of a 96-well flat bottom black polystyrene plate (Corning Life Sciences, Corning, NY, USA) and the plate was placed into the fluorescence spectrophotometer. The plate was then shaken for 5 s and the samples were read once every 30 s for a total of 3 min to establish a peak fluorescent measurement for all samples. The plate was then removed from the fluorescence spectrophotometer and buffer only or purified Mical^Redox^ protein (final concentration 50 nM or 100 nM was added for these experiments but more or less can be added, not shown) and NADPH (MP Biomedicals, Santa Ana, CA, USA) (final concentration 100 µM was added for these experiments but more or less can be added, not shown) were added into the wells. The plate was then returned to the spectrophotometer. It should be noted that after adding purified Mical^Redox^ protein and the NADPH co-enzyme into the wells, the plate should be immediately returned in the spectrophotometer for reading. If the fluorescence of the samples in the 96-well plate is not being read immediately, the reading for initial actin disassembly activity by Mical^Redox^ protein will be missed. Also note that since FAD is purified with Mical^Redox^ protein (it is non-covalently attached), there is no need to add additional FAD. The plate was then shaken for 5 s and the samples in each well were read once every 30 s for a total of 60 min. The depolymerization of the pyrene-labeled actin was observed as a decrease in the fluorescent signal over time. The data from the fluorescence spectrophotometer was then exported and after normalizing the zero points of all samples, graphs were drawn using GraphPad Prism (San Diego, CA, USA).

## Data Availability

All data generated or analysed during this study are included in this published article and its Supplementary Materials. All materials, data and associated protocols will be made promptly available to others without preconditions.

## Supplementary Materials

The following are available online at https://www.mdpi.com/1422-0067/22/4/1991/s1, Figure S1: Further analyses of Mical^Redox^ protein expression and purification; Figure S2: Further analyses of Mical^Redox^ protein’s catalytic and F-actin disassembly activity.
