# Supplementary material for: Enhanced Production of the Mical Redox Domain for Enzymology and F-actin Disassembly Assays
Source: Int J Mol Sci. 2021 Feb 17;22(4):1991. doi: 10.3390/ijms22041991 (PMC7922515; doi:10.3390/ijms22041991)
Supplement: Supplementary file 1 [file ijms-22-01991-s001.pdf]

Supplementary Material for

Enhanced Production of the Mical Redox Domain for Enzymology and F-actin Disassembly Assays

Jimok Yoon[+], Heng Wu[+], Ruei-Jiun Hung and Jonathan R. Terman*

+ These authors contributed equally

* Correspondence: author: jonathan.terman@utsouthwestern.edu; Tel: 214-648-1464; Fax: 214-648-1801

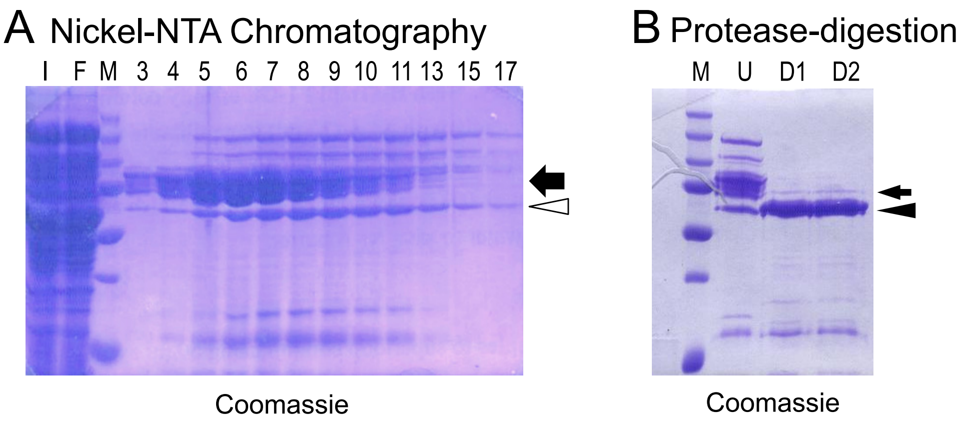

Figure S1. Further analyses of Mical^{Redox} protein purification. (A-B) Unlike the expression and purification strategies we developed that allowed us to obtain high amounts of protein for the RedoxCH portion of the *Drosophila* and human MICALs [1-4], these strategies did not prove useful for expression and purification of high amounts of the Mical^{Redox} protein. In particular, in this example, similar to our strategy for expressing and purifying the RedoxCH portion of the *Drosophila* and human MICALs [3, 4], we used bacterial cells engineered to express cold-adapted chaperonin proteins Cpn60 and co-chaperonin Cpn10 from the cryophilic bacterium, *Oleispira antarctica* [5], and induction temperatures as low as 4–12°C. We also used the pET43.1bNG vector [6], which has a Nus solubility tag and has been used extensively to obtain purified active Mical^{RedoxCH} protein [2, 4, 7-11]. However, in the case of Mical^{Redox} protein, we found that although high levels of solubility-tagged Mical^{Redox} protein was expressed (A, large arrow), we could not obtain appreciable levels of Mical^{Redox} protein (B, arrow) following digestion with a protease to remove the solubility tag. Further, in the case of the Nus solubility-tagged Mical^{Redox} protein, additional analysis indicated that Mical^{Redox} protein became destabilized and precipitated from the solution after removal of the Nus solubility tag from the Mical^{Redox} protein (see Figure 2B). Cpn60 (open arrowhead in A), Nus solubility tag (arrowhead in B). I, input; F, flow-through; M, protein markers; 3-17 (different fractions eluting from the Nickel-NTA column); U (undigested), D1 (thrombin digested at room temperature); D2 (thrombin digested at 4°C).

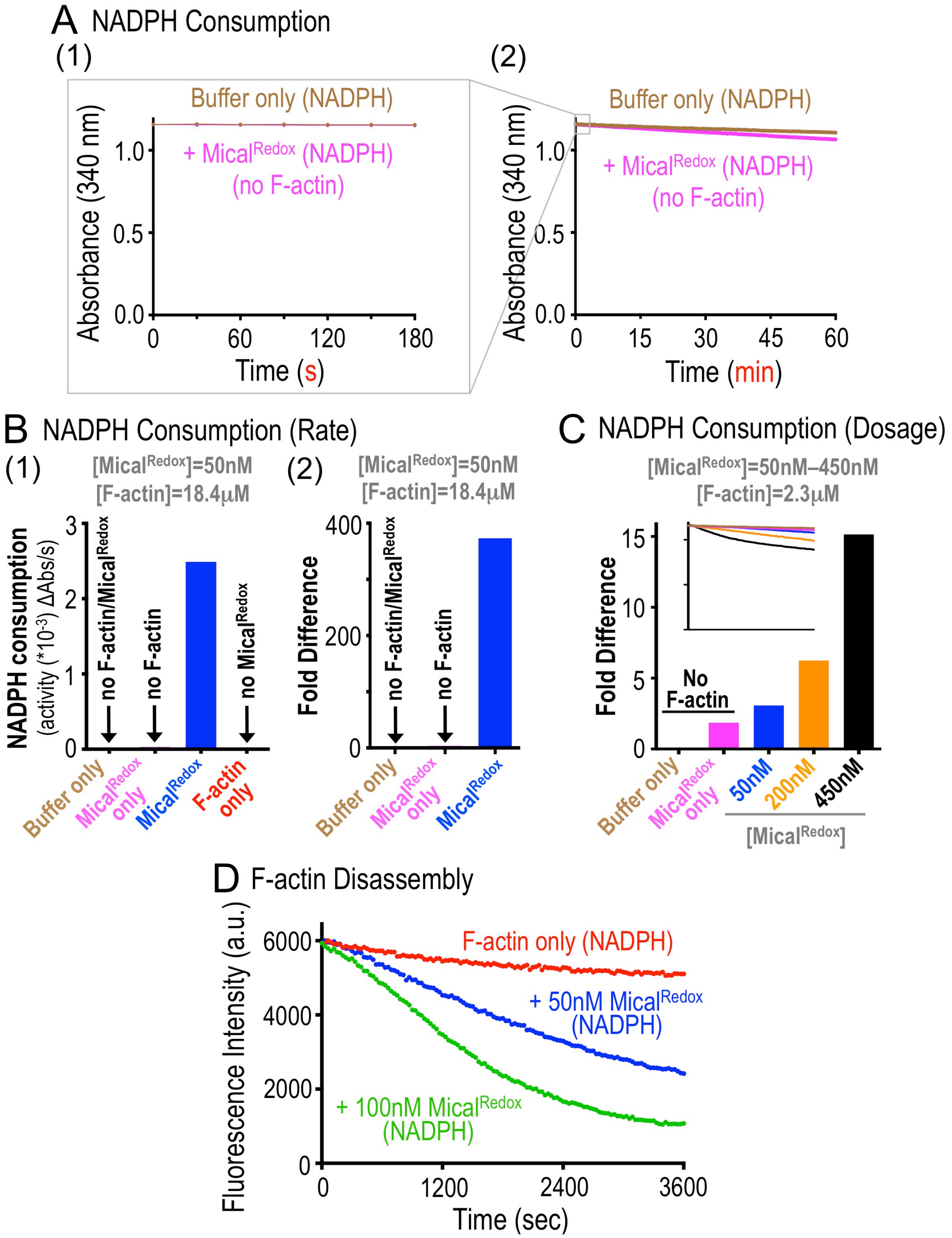

Figure S2. Further analyses of Mical^{Redox} protein's catalytic and F-actin disassembly activity. (A-C) NADPH consumption assays as in Figure 4B and where NADPH is present in all conditions. (A) NADPH consumption activity of Mical^{Redox} protein on its own (without its F-actin substrate). Our newly purified Mical^{Redox} protein has enzyme activity (Figure 4B). Furthermore, similar to what we have previously reported for Mical^{RedoxCH} protein (e.g., [4, 7]), Mical^{Redox} protein on its own (without F-actin) has little NADPH consumption activity (2), but Mical's substrate F-actin substantially increases Mical's NADPH consumption activity (e.g., compare the graph in (1) and note that it is at the same timescale as Figure 4B, but with substantially less NADPH consumption). [Mical^{Redox}] = 50 nM as in Figure 4B. [NADPH] = 200 μM. Time = 180 seconds (s) in (1) and 60 minutes (min) in (2). (B) Rate of NADPH consumption. (1) Mical^{Redox} protein's calculated rate of NADPH consumption. A similar activity for the Mical^{Redox} protein was reported in [11] and corresponds in general to that seen for the Mical^{RedoxCH} protein [4, 7]. (2) The fold difference in the NADPH consumption (change of Absorption at 340 nm) for the different conditions versus F-actin only. NADPH present

in all conditions. [$Mical^{Redox}$] = 50 nM. [NADPH] = 200 μM. [F-actin] = 18.4 μM as in Figure 4B. **(C)** $Mical^{Redox}$ protein dosage-dependent differences in NADPH consumption. $Mical^{Redox}$ protein shows a dosage dependent increase in enzymatic activity such that it consumes more NADPH as it is added in higher concentrations. Note also that as the F-actin concentration is increased, the NADPH consumption of a given [$Mical^{Redox}$] substantially increases (e.g., compare the blue bars ([50 nM]) in C to B (2)). NADPH present in all conditions. Buffer only and $Mical^{Redox}$ only do not contain F-actin as in A and B. [NADPH] = 200 μM; [F-actin] = 2.3 μM. [$Mical^{RedoxCH}$] is as indicated in each bar of the graph, which is also color-coded to the inset graph (change in absorbance (340 nm) as in A). **(D)** Our newly purified $Mical^{Redox}$ protein has F-actin disassembly activity (Figure 4D). Furthermore, using Pyrene-actin depolymerization assays as in Figure 4D (where the fluorescence of polymerized actin decreases as F-actin depolymerizes) and similar to what we have previously reported for $Mical^{RedoxCH}$ protein (e.g., [2, 4, 7]), we find that as the levels of our new $Mical^{Redox}$ protein increases, F-actin disassembly increases. [$Mical^{Redox}$] as indicated = 50 nM or 100 nM; [NADPH] = 100 μM; [F-actin] = 4.65 μM.

Supplementary References

1. Gupta, N.; Terman, J. R., Characterization of MICAL flavoprotein oxidoreductases: Expression and solubility of different truncated forms of MICAL. In *Flavins and Flavoproteins 2008 (Proceedings of the 16th International Symposium on Flavins and Flavoproteins)*, Frago, S.; Gomez-Moreno, C.; Medina, M., Eds. Prensas Universitarias De Zaragoza: Zaragoza, 2008; pp 345-350.
2. Hung, R. J.; Yazdani, U.; Yoon, J.; Wu, H.; Yang, T.; Gupta, N.; Huang, Z.; van Berkel, W. J.; Terman, J. R., Mical links semaphorins to F-actin disassembly. *Nature* **2010,** *463*, 823-7.
3. Wu, H.; Hung, R. J.; Terman, J. R., A simple and efficient method for generating high-quality recombinant Mical enzyme for in vitro assays. *Protein Expr. Purif.* **2016,** *127*, 116-24.
4. Wu, H.; Yesilyurt, H. G.; Yoon, J.; Terman, J. R., The MICALs are a Family of F-actin Dismantling Oxidoreductases Conserved from Drosophila to Humans. *Sci. Rep.* **2018,** *8*, 937.
5. Ferrer, M.; Chernikova, T. N.; Yakimov, M. M.; Golyshin, P. N.; Timmis, K. N., Chaperonins govern growth of Escherichia coli at low temperatures. *Nat. Biotechnol.* **2003,** *21*, 1266-7.
6. Gupta, N.; Wu, H.; Terman, J. R., Data presenting a modified bacterial expression vector for expressing and purifying Nus solubility-tagged proteins. *Data Brief* **2016,** *8*, 1227-31.
7. Hung, R. J.; Pak, C. W.; Terman, J. R., Direct redox regulation of F-actin assembly and disassembly by Mical. *Science* **2011,** *334*, 1710-3.
8. Hung, R. J.; Spaeth, C. S.; Yesilyurt, H. G.; Terman, J. R., SelR reverses Mical-mediated oxidation of actin to regulate F-actin dynamics. *Nat. Cell Biol.* **2013,** *15*, 1445-54.
9. Grintsevich, E. E.; Ge, P.; Sawaya, M. R.; Yesilyurt, H. G.; Terman, J. R.; Zhou, Z. H.; Reisler, E., Catastrophic disassembly of actin filaments via Mical-mediated oxidation. *Nat. Commun.* **2017,** *8*, 2183.
10. Grintsevich, E. E.; Yesilyurt, H. G.; Rich, S. K.; Hung, R. J.; Terman, J. R.; Reisler, E., F-actin dismantling through a redox-driven synergy between Mical and cofilin. *Nat. Cell Biol.* **2016,** *18*, 876-885.
11. Yoon, J.; Kim, S. B.; Ahmed, G.; Shay, J. W.; Terman, J. R., Amplification of F-Actin Disassembly and Cellular Repulsion by Growth Factor Signaling. *Dev. Cell* **2017,** *42*, 117-129.
